# You have been QUALIFIED for a smokeless e-cig starter kit

**DOI:** 10.1136/jech-2014-203879

**Published:** 2014-04-29

**Authors:** Kate Hunt, Helen Sweeting

**Affiliations:** 1 MRC/CSO Social & Public Health Sciences Unit, Institute of Health and Well Being, Glasgow University, Glasgow, UK; 2 MRC Social and Public Health Sciences Unit, Glasgow, UK

**Keywords:** Smoking, Gender, Public Health

**Figure 1:**
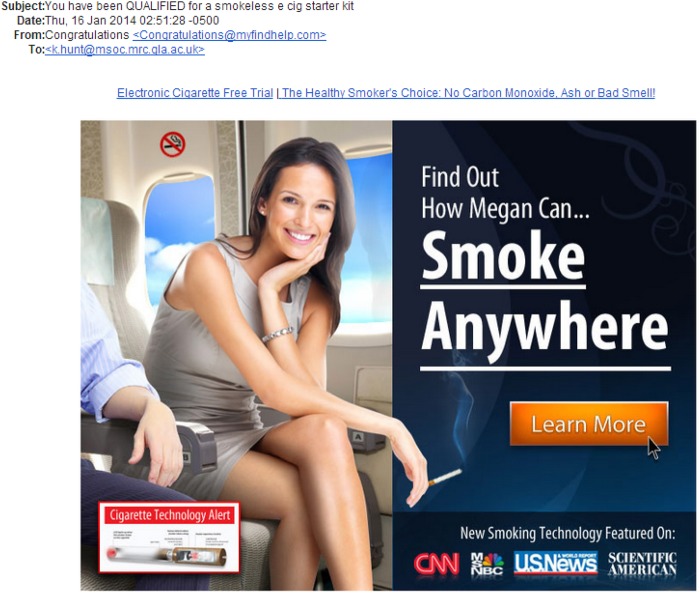
Screenshot of unsolicited email.

Shortly after the shock of seeing e-cigarette adverting on television, an unsolicited e-mail arrived promoting an ‘e-cig starter kit’ (figure 1). This showed ‘Megan’ (attractive, slim, elegant, professional, confident and happy) ‘smoking’ an e-cigarette, apparently on a plane. Incongruously, the e-cigarette billows smoke. The sender's address and titles of embedded links suggest the ease of trying e-cigarettes, and that e-cigarettes are healthy and inoffensive. Ingeniously, the advert can be read as showing that holding a cigarette object is attractive and socially desirable, *and* that e-cigarettes are (somewhat) distinct from ‘ordinary’ cigarettes.

Emerging research raises concerns over whether e-cigarettes renormalise and reglamourise smoking and/or act as a gateway to smoking.^1^ ^2^ Within present legislation, ‘Megan’ can ‘smoke’ her e-cigarette in public spaces because e-cigarettes are not subject to smoke-free regulation. They can also be advertised, although some may question whether a *smoking* e-cigarette complies with guidelines.

In 2013, US Democratic Congress members wrote to e-cigarette manufacturers regarding marketing tactics likely to ‘hook’ young people,^3^ and posted a presentation highlighting parallels with earlier cigarette marketing.^4^ As gender and health researchers, we also note the strong resemblance to images of women in adverting which so successfully drew earlier generations of women to smoking.^5–7^
